# Challenges in Caring for Linguistic Minorities in the Pediatric Population

**DOI:** 10.3390/children6080087

**Published:** 2019-07-25

**Authors:** Logan DeBord, Kali Ann Hopkins, Padma Swamy

**Affiliations:** 1 Department of Dermatology, School of Medicine, University of Colorado, Anschutz Cancer Pavilion Building, 3rd Floor, 1665 Aurora Court, Aurora, CO 80045, USA; 2 Department of Pediatrics, Feinberg School of Medicine, Northwestern University, 225 E Chicago Ave, Chicago, IL 60611, USA; 3 Department of Pediatrics, Baylor College of Medicine, 8080 N. Stadium Drive, Houston, TX 77054, USA

**Keywords:** limited English proficiency (LEP), linguistic minorities, medical interpreters, immigrants, refugees, Mayan

## Abstract

Physicians in the United States (U.S.) face unique obstacles in providing care for persons with limited English proficiency (LEP), especially speakers of rare languages. Lack of professional resources is not a problem exclusive to health care delivery, with speakers of Mayan dialects receiving increasingly narrow representation in detention centers and immigration courts at the U.S.–Mexico border. Parent-child dynamics and other crucial information related to pediatric care may be lost in translation in the event of inadequate interpreter services. Several strategies could address disparities in medical care faced by persons with LEP, speaking rare as well as more common languages. These include increasing the availability of professional interpreters via expanded and/or incentivized training programs, providing focused education in interpreter services for medical students, and unifying interpretation services provided by local consulates and nonprofit agencies for both medical and legal purposes.

## 1. Introduction

The current United States (U.S.) population was built on the foundations of a rich immigration history. While English remains the unofficial language in practice, approximately 21.6% of U.S. residents ages 5 and older speak another language primarily at home, according to the U.S. Census [1]. An executive order released in 2000 mandated that federal agencies improve systems and programs for Limited English Proficiency (LEP) individuals; in 2003, the Department of Health and Human Services (HHS) published guidelines on how to address the order [2,3]. The HHS required that health care entities take “reasonable steps to provide meaningful access to each individual with limited English proficiency eligible to be served or likely to be encountered in its health programs and activities” [4]. According to Jacobs et al., there are no federal guidelines requiring certified or licensed interpreters. While not mandated, HHS does encourage the use of certified medical interpreters [2]. In the U.S., there are two accreditation bodies that provide certification for medical interpreters: the National Board of Certification for Medical Interpreters and the Certification Commission for Healthcare Interpreters [2]. However, interpretation is costly and sometimes difficult to implement in small clinics which essentially function as businesses [4]. However, several state-run versions of our federal insurance program will reimburse health care/language services for use of interpreters [4].

While this provision broadly refers to all interpretation, we encountered two situations in which the existing system to connect patients with trained interpreters failed. While pantomiming or using ad hoc interpreters may be tempting in the setting of a busy teaching environment, one meta-analysis showed that quality of care for limited English proficient patients is inferior with the use of such untrained interpreters [5]. Lack of proficiency in a patient’s native language commonly results in errors of medical interpretation, with potential clinical consequences [6]. Even Spanish-speaking patients in the United States have been found to suffer reduced clinical encounter time and substandard treatment compared to native English speakers [7]. However, language services are legally mandated to be available free-of-charge to patients in facilities that receive federal funding. The first situation that we encountered (described by L.D.) details a challenge in caring for a speaker of a rare language despite having a fairly robust interpretation service program in place. The second situation (described by K.A.H.) gives insight into a common practice by providers when faced with language difficulties and was encountered in a resource-limited setting outside the U.S.

## 2. A Shortage of Qualified Interpreters in Rare Languages (L.D.)

I was a third-year medical student conducting vaccinations at a children’s mobile clinic that delivers care to patients who are often under- or uninsured. Most of these patients were recipients of Medicaid, which is our combined federal and state government insurance program that addresses medical costs for those with low income. Many patients in our mobile clinic were also recent immigrants to the U.S. with limited English proficiency. Such was the case when a newly arrived Guatemalan child presented to the clinic with his father, who spoke their primary language of an uncommon Mayan dialect in addition to very rudimentary Spanish. This problem compounded unravelling the child’s chief complaint of headache for our health care team. 

Having rotated extensively in our county hospital, I was confident in my ability to have a Spanish translator, either in person or via phone, at a patient’s bedside within minutes. However, medical interpreters in rare languages are more difficult to find. Often a scheduled appointment is required weeks in advance. Our team resorted to relying on the father’s broken Spanish, which was then translated into English via an on-demand Spanish phone interpreter. However, I noted some discomfort in the boy’s responses to our probing questions: had he seen anything disturbing during the journey? Was he satisfied with the meals that his father was able to provide?

Additionally, it also became apparent that not all of our questions were being understood by the father once translated from English to Spanish. My attending physician (P.S.) decided that on this visit, our priority should be to screen for *red flag* symptoms of headache that might signal intracranial pathology. We were sufficiently comfortable in our determination via interview that the headaches were not occipital in location, did not awaken the child from sleep, and were not accompanied by nausea, vomiting, or ataxia. We also completed a neurological exam, which revealed the absence of focal weakness, papilledema and nuchal rigidity. My attending physician ultimately decided to schedule an interpreter in their native language and reassess the situation at a follow-up visit in two weeks. I left clinic that day wondering about the factors contributing to the child’s suffering. Would fluency in his native tongue have allowed us to pick up on nuances that might be key to the diagnosis?

This recent interaction with the father and his son resulted in my becoming aware of the obstacles in providing care for persons with limited English proficiency in my home country. Lack of professional resources for persons with LEP is not a problem exclusive to health care delivery, with speakers of Mayan dialects also receiving increasingly narrow representation in detention centers and immigration courts at our Southern border [8].

## 3. Risks of Playing the Telephone Game with Medical Information (K.A.H.)

As an Internal Medicine-Pediatrics resident rotating at a teaching hospital in Kenya, I arrived in a setting in which trained interpreters do not exist. My team was rounding in the “failure-to-thrive room,” which was slightly bigger than a closet and crowded with five patients and their mothers. During rounds, we diagnosed a child with visceral leishmaniasis, but his mother spoke only a rare tribal language. Fortunately, another mother in the room also spoke this language, and we found a nursing student who spoke this second mother’s primary tribal language, in addition to Swahili. In the end, the team translated my English to Swahili, and then to both tribal languages. Aware of the potential pitfalls of *playing telephone* with medical information, I shortened the message to the diagnosis, its method of transmission, the prognosis, and treatment. The mother eventually nodded in understanding that her son would likely recover, as long as he could receive antibiotics in the hospital for an additional three weeks.

## 4. Options to Address Shortcomings in Care for U.S. Residents with Limited English Proficiency

While not ideal, interactions such as these may avoid the consequence of minimal understanding for patients in a resource-limited setting. However, inherent structural and attitudinal barriers underlie a lack of access to qualified interpreters for both Spanish speakers and speakers of rare languages alike in the United States. Our country currently lacks a required standardized training and certification process for medical interpreters, which increases the likelihood of preventable medical errors in the care of such patients [9]. The rigors of medical training leave little time for trainees to become proficient in another language, despite many being surrounded by non-English speakers during clinical rotations. In medical schools, there is widespread instruction on the need to always use a qualified interpreter but seldom advanced discussions regarding how to advocate for linguistic minorities. As we have learned, essential parent-child dynamics, as well as patient-provider rapport, may also be lost in translation when playing games of *medical telephone*.

Several possible solutions can address disparities in care faced by both patients and parents with LEP. Increasing the number and availability of professional interpreters is paramount to minimizing clinical consequences for such minorities. One study supports the notion that requiring at least 100 training hours for interpreters would likely significantly reduce errors in medical interpretation [10]. From our personal experience, we observed that patients generally seemed more satisfied when speaking to an interpreter visible via videoconference compared to telephone, a finding which was reflected in a previous study [9]. In terms of medical students’ competency in using available interpreter services, focused education likely results in earlier proficiency compared to those who receive no training [11]. Incorporating translator difficulties, particularly in pediatric encounters in which there is both the perspective of the patient and guardian to consider, into patient simulations in medical training might be beneficial to prepare for real-life scenarios.

For rare languages in particular, outsourcing hospital-based medical interpretation to call centers in foreign countries may be a key strategy to provide access independent of the patient’s location [12]. However, a more pragmatic method might be to address problems in our health care and legal systems simultaneously. Increasing Mayan-language interpreter courses could bolster the number of qualified interpreters at a time when demand is expected to increase in both immigration courts, safety net clinics, and other community settings [13,14]. Unifying interpretation services provided by local consulates and nonprofit agencies for both medical and legal purposes could ensure that speakers of uncommon Central American dialects receive adequate representation.

Pediatricians in training should be aware that difficulties in interpretation can add to the challenge of untangling already-complex relationships between the patient and guardian. In our patient encounter at the mobile clinic, we were aware of the possibility that the boy might not be willing to admit that he was hungry or thirsty in front of his father. Ready accessibility of an interpreter in the patient’s primary language is even more important in these types of scenarios. When this is not possible, efforts should be made to rule out life-threatening etiologies of the chief complaint while scheduling adequate follow-up with a qualified interpreter. 

Educating medical students to the breadth of challenges faced by linguistic minorities via clinical exposure, didactics by certified interpreters, and patient simulations would prepare them to advocate for these patients in their future careers. Simultaneously, local and federal agencies should mandate a standardized certification process for interpreters and implement incentives for entry into the field. This dual approach would ensure that pediatric patients have their voices heard in an increasingly global environment.
